# Use of Machine Learning to Estimate the Per-Protocol Effect of Low-Dose Aspirin on Pregnancy Outcomes: A Secondary Analysis of a Randomized Clinical Trial

**DOI:** 10.1001/jamanetworkopen.2021.43414

**Published:** 2022-03-09

**Authors:** Yongqi Zhong, Maria M. Brooks, Edward H. Kennedy, Lisa M. Bodnar, Ashley I. Naimi

**Affiliations:** 1 Department of Epidemiology, The Johns Hopkins University, Baltimore, Maryland; 2 Department of Epidemiology, University of Pittsburgh, Pittsburgh, Pennsylvania; 3 Department of Data Science and Statistics, Carnegie Mellon University, Pittsburgh, Pennsylvania; 4 Department of Epidemiology, Emory University, Atlanta, Georgia

## Abstract

**Importance:**

In randomized clinical trials (RCTs), per-protocol effects may be of interest in the presence of nonadherence with the randomized treatment protocol. Using machine learning in per-protocol effect estimation can help avoid model misspecification owing to strong parametric assumptions, as is common with standard methods (eg, logistic regression).

**Objectives:**

To demonstrate the use of ensemble machine learning with augmented inverse probability weighting (AIPW) for per-protocol effect estimation in RCTs and to evaluate the per-protocol effect size of aspirin on pregnancy.

**Design, Setting, and Participants:**

This secondary analysis used data from 1227 women in the Effects of Aspirin in Gestation and Reproduction (EAGeR) trial, a multicenter, block-randomized, double-blind, placebo-controlled clinical trial of the effect of daily low-dose aspirin on pregnancy outcomes in women at high risk of pregnancy loss. Participants were recruited at 4 university medical centers in the US from June 15, 2007, to July 15, 2012. Women were followed up for 6 menstrual cycles for attempted pregnancy and 36 weeks of gestation if pregnancy occurred. Follow-up was completed on August 17, 2012. Data analyses were performed on July 9, 2021.

**Exposures:**

Daily low-dose (81 mg) aspirin taken at least 5 of 7 days per week for at least 80% of follow-up time relative to placebo.

**Main Outcomes and Measures:**

Pregnancy detected using human chorionic gonadotropin (hCG) levels.

**Results:**

Among the 1227 women included in the analysis (mean SD age, 28.74 [4.80] years), 1161 (94.6%) were non-Hispanic White and 858 (69.9%) adhered to the protocol. Five machine learning models were combined into 1 meta-algorithm, which was used to construct an AIPW estimator for the per-protocol effect. Compared with adhering to placebo, adherence to the daily low-dose aspirin protocol for at least 5 of 7 days per week was associated with an increase in the probability of hCG-detected pregnancy of 8.0 (95% CI, 2.5-13.6) more hCG-detected pregnancies per 100 women in the sample, which is substantially larger than the estimated intention-to-treat estimate of 4.3 (95% CI, −1.1 to 9.6) more hCG-detected pregnancies per 100 women in the sample.

**Conclusions and Relevance:**

These findings suggest that a low-dose aspirin protocol is associated with increased hCG-detected pregnancy in women who adhere to treatment for at least 5 days per week. With the presence of nonadherence, per-protocol treatment effect estimates differ from intention-to-treat estimates in the EAGeR trial. The results of this secondary analysis of clinical trial data suggest that machine learning could be used to estimate per-protocol effects by adjusting for confounders related to nonadherence in a more flexible way than traditional regressions.

**Trial Registration:**

ClinicalTrials.gov Identifier: NCT00467363

## Key Points

**Question:**

How can machine learning be used to estimate per-protocol effects in randomized clinical trials?

**Findings:**

In a cohort of 1227 women derived from secondary analysis of a randomized clinical trial, ensemble machine learning with augmented inverse probability weighting was used to estimate the per-protocol effect of daily low-dose aspirin on pregnancy detected using human chorionic gonadotropin (hCG) levels. Relative to placebo, adherence to the assigned treatment protocol was associated with an increase of 8.0 hCG-detected pregnancies per 100 women, approximately double the intention-to-treat estimates.

**Meaning:**

These findings suggest that in per-protocol analysis, machine learning techniques may allow for confounder adjustment while reducing the occurrence of model misspecification.

## Teaser

This secondary analysis of a randomized clinical trial uses machine learning methods to estimate the per-protocol effects of a low-dose aspirin regimen on pregnancy in the Effects of Aspirin in Gestation and Reproduction trial.

## Introduction

Intention-to-treat (ITT) effects from randomized clinical trials (RCTs) are the reference standard for evaluating treatment effects. Importantly, ITT effects capture the impact of assigning treatments to individuals. The ITT approach does not provide estimates of the effects that would be observed if all individuals adhered with a desired treatment protocol^1,2,3^—that is, in the presence of nonadherence, the ITT effects may differ in important ways from the effect of taking the treatment under study in a specified way (ie, a study protocol).^2,4^

Several investigators^2,5^ have called for a more formal approach to per-protocol effect estimation in RCTs, and several per-protocol analyses^4,6,7,8,9,10,11,12^ have demonstrated important deviations from ITT estimates when nonadherence is accounted for. Unfortunately, when per-protocol effects are targeted in RCTs, all limitations associated with observational studies must be considered, such as confounding bias.^2,4^ Machine learning methods can be used with augmented inverse probability weighting (AIPW) and stacked regression models to overcome some of these limitations^13,14^ and to estimate per-protocol effects when adjusting for confounding variables. However, compared with traditional regression models, machine learning methods may be better suited to avoiding problems with model misspecification.^15,16^ For example, a model would be misspecified if a linear regression were used to fit 2 variables with nonlinear relations (eg, perinatal mortality and maternal hemoglobin levels).^17^ Many machine learning algorithms can avoid these problems,^15,16^ but they have not yet been applied to scenarios in which per-protocol effects are of primary interest.

In this report, we illustrate the use of machine learning methods to estimate the per-protocol effects of low-dose aspirin on pregnancy in the Effects of Aspirin in Gestation and Reproduction (EAGeR) trial. We evaluate how machine learning methods can be used to estimate per-protocol effects and discuss the feasibility and trade-offs of using machine learning methods for adherence-adjusted analyses.

## Methods

### Study Design

In this secondary analysis of an RCT, we used the data from the EAGeR trial—a multicenter, block randomized, double-blind, placebo-controlled clinical trial. The EAGeR trial recruited women aged 18 to 40 years who were actively trying to become pregnant and who had 1 or 2 prior pregnancy losses and no history of infertility from 4 university medical centers in the US from June 15, 2007, to July 15, 2012. Follow-up was completed on August 17, 2012. A total of 1228 women were recruited and randomized. Most of the participants (1161 [94.5%]) self-identified as non-Hispanic White race and ethnicity. For as many as 6 menstrual cycles, participants were followed up biweekly in their first 2 cycles and monthly afterward while attempting pregnancy. If a pregnancy was observed, follow-up continued throughout pregnancy for the live birth outcome (the registered primary end point of the trial). Institutional review board approvals at each clinical site and data coordinating center were obtained. A data safety and monitoring board was also formed to ensure participants’ safety and monitor the efficacy of the trial. Missing data were addressed via single imputation. Details about study design, eligibility criteria, baseline characteristics, and other relevant information has been published elsewhere,^4,18,19,20^ and a complete copy of the trial protocol is available in Supplement 1. All participants provided written informed consent. The present analysis was conducted on July 9, 2021, and this study followed the Consolidated Standards of Reporting Trials (CONSORT) reporting guideline.

### Treatment and Adherence

With 1:1 randomized treatment allocation, the treatment group (n = 615) received preconception-initiated daily low-dose aspirin (81 mg) plus folic acid (400 μg) and the control group (n = 613) received placebo plus folic acid (400 μg). For women who became pregnant, study treatment was to continue until week 36 of gestation. A total of 1227 women were included in the analysis of the present study (1 participant had missing follow-up data).

Adherence was assessed via bottle weight measurements in both groups during regular follow-up visits. Weekly adherence status was determined by evaluating whether a participant took their assigned pills for at least 5 of 7 days (equivalent to 70%) during a given week. A woman was deemed adherent with the study protocol if, in any given week during follow-up, she took a pill on at least 5 of 7 days. For each woman, this time-varying measure was categorized as adherent if the mean adherence during follow-up was at least 80% of their follow-up time before becoming pregnant or the entire follow-up time for those without pregnancy. Notably, this adherence status is a dichotomized, time-fixed variable, which is commonly used in a typical per-protocol analysis but differs from the previous analyses of this trial.^4^

### Outcome

Pregnancy detected with human chorionic gonadotropin (hCG) levels during the defined treatment period was the primary outcome for this analysis. Pregnancies were determined by a positive result on a real-time hCG pregnancy test (QuickVue; Quidel), which was sensitive to 25 mIU/mL of hCG. The test was conducted at each study visit when expected menses were absent or by batched urine testing using daily first morning urine collected at home, stored on the last 10 days of each participant’s first cycle after randomization, and analyzed in the laboratory.

### Baseline Covariates and Postrandomization Confounders

Baseline data on demographic, behavioral, and pregnancy history information were obtained via questionnaires, including age, race and ethnicity, educational level, marital status, income, frequency of exercise, alcohol and cigarette use in the past year, number of prior pregnancy losses, and number of months attempting pregnancy before randomization. Physical measurements of height and weight were used to calculate body mass index at baseline. Blood samples were also collected to measure serum high-sensitivity C-reactive protein levels using an immunoturbidimetric assay (COBAS 6000 autoanalyzer; Roche Diagnostics) with a detection limit of 0.0015 mg/dL (to convert to mg/L, multiply by 10).

Postrandomization confounders, including unusual (or excessive) bleeding and nausea and/or vomiting, were collected via questionnaire at regular intervals during follow-up. Similar to overall adherence status, we dichotomized these 2 postrandomization confounders by setting the values to 1 if a woman experienced unusual bleeding for at least 50% or nausea and/or vomiting for at least 20% of their follow-up time at least 1 of 7 days (20%) per week.

### Statistical Analysis

In this study, we selected a protocol in which women would adhere to their assigned treatment for at least 5 of 7 days of a given week and for more than 80% of their follow-up time before pregnancy. This protocol allows us to evaluate whether consistently taking aspirin (vs placebo) is associated with an increased probability of experiencing an hCG-detected pregnancy. Differences in treatment, outcome, baseline characteristics, and postrandomization confounders between adherence status were tested by χ^2^ test for categorical variables and by Kruskal-Wallis test for continuous variables. To examine the impact of different adherence thresholds on the overall findings as well as the number of individuals who were adherent and nonadherent with treatment in the samples, we explored protocols under assigned treatment for at least 4 of 7 days, 5 of 7 days, and 6 of 7 days of a given week for 60%, 70%, and 80% of person-weeks of follow-up.

Our target per-protocol effect is defined as the average per-protocol treatment effect among women who adhered to the aspirin protocol.^21^ To estimate this per-protocol effect of interest with machine learning methods, we used an AIPW estimator with an ensemble machine learner known as the Super Learner (or stacked generalization).^16,22,23,24^ Per-protocol effects were quantified on both the risk difference and the risk ratio scales for the pregnancy outcome.

Stacking is a machine learning technique that combines several different algorithms into a single meta-algorithm. The benefit of using stacking as opposed to a single regression model or machine learning algorithm (eg, the least absolute shrinkage and selection operator regression or random forests) is flexibility; stacking algorithms can combine the strengths of each individual algorithm based on how they fit the data, thus avoiding the need of the potentially strong assumptions on which single algorithms rely for validity. The stacking technique first trains several machine learning models individually as the first layer. Estimates (or predictions) of the individual models from the first layer are then used as the input for the second layer, which is the meta-algorithm. Cross-validation is used to determine the importance of each first-layer algorithm in the overall meta-algorithm and to avoid potential overfitting.^16,23^

In this study, we stacked 5 regression models (from traditional to flexible): a standard generalized linear model with main effects only, a standard generalized linear model with main effects and all 2-way interactions, multivariate adaptive regression splines,^25^ random forests,^26^ and extreme gradient boosting.^27^ For multivariate adaptive regression splines, random forests, and extreme gradient boosting, a grid of tuning parameters was included in the stacking algorithm. All algorithms were combined into the meta-algorithm via nonnegative least squares. The predictions from these stacked models were then used to construct the AIPW estimator.

Augmented inverse probability weighting is a “doubly robust” estimator that relies on estimating the exposure model (ie, propensity score) and the outcome model separately (both modeled with the stacking algorithm) and then combining the predictions from these models into a single estimator that quantifies the average treatment effect.^22^ Augmented inverse probability weighting is consistent as long as at least the exposure model or the outcome model is correctly specified. Further, AIPW performs well, even when using flexible machine learning methods.^13,24^ Using the aforementioned stacked machine learning algorithm, we estimated propensity scores by modeling the exposure with the aforementioned baseline covariates (exposure model) and constructed the outcome model using the exposure and those covariates. Cross-fitting, an additional layer of the fitting process on top of the stacking machine learning, is applied in the AIPW estimator to obtain valid inference (eg, low bias) and to further avoid overfitting.^13,24^

Sensitivity analyses were conducted by using other thresholds of time-fixed adherence status, which is a combination of adherence to at least 4, 5, and 6 days in a given week during at least 60%, 70%, and 80% of person-weeks of follow-up. In addition, we also provided the ITT estimates obtained via g-computation,^28^ inverse probability weighting,^29^ targeted maximum likelihood estimation,^30^ and AIPW and unadjusted per-protocol effects (with different thresholds) estimated by g-computation, inverse probability weighting, targeted maximum likelihood estimation, and AIPW. We constructed g-computation and inverse probability weighting with a standard generalized linear model with main effects only. Targeted maximum likelihood estimation is also a doubly robust estimator, which performs well when machine learning methods are used. We constructed the targeted maximum likelihood estimator using the same stacking machine learning algorithms for the AIPW. Further, we repeated all analyses after adjusting for postrandomization confounders (ie, unusual bleeding and nausea and/or vomiting).

All analyses were performed in R, version 3.6.2 (R Project for Statistical Computing). We conducted the implemented AIPW estimation using the AIPW package. The AIPW package supports the Super Learner package for stacking machine learning with cross-validation and provides a user-friendly interface for cross-fitting.^24,31^ A prior study using the data resampled from the EAGeR trial^24^ has shown excellent statistical performance for the AIPW package. Targeted maximum likelihood estimation was conducted with the tmle package.^32^ The code needed to reproduce our analyses is available in the eMethods in Supplement 1. Two-sided *P* < .05 indicated statistical significance.

## Results

**Figure 1.**
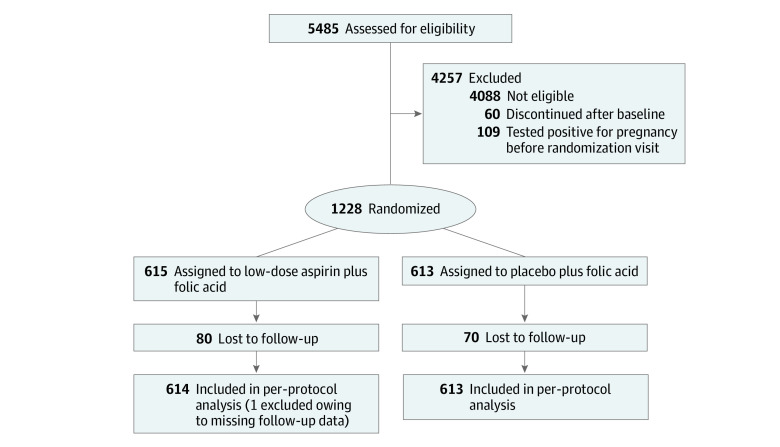
CONSORT Study Flow Diagram for the Effects of Aspirin in Gestation and Reproduction (EAGeR) Trial

**Figure 2.**
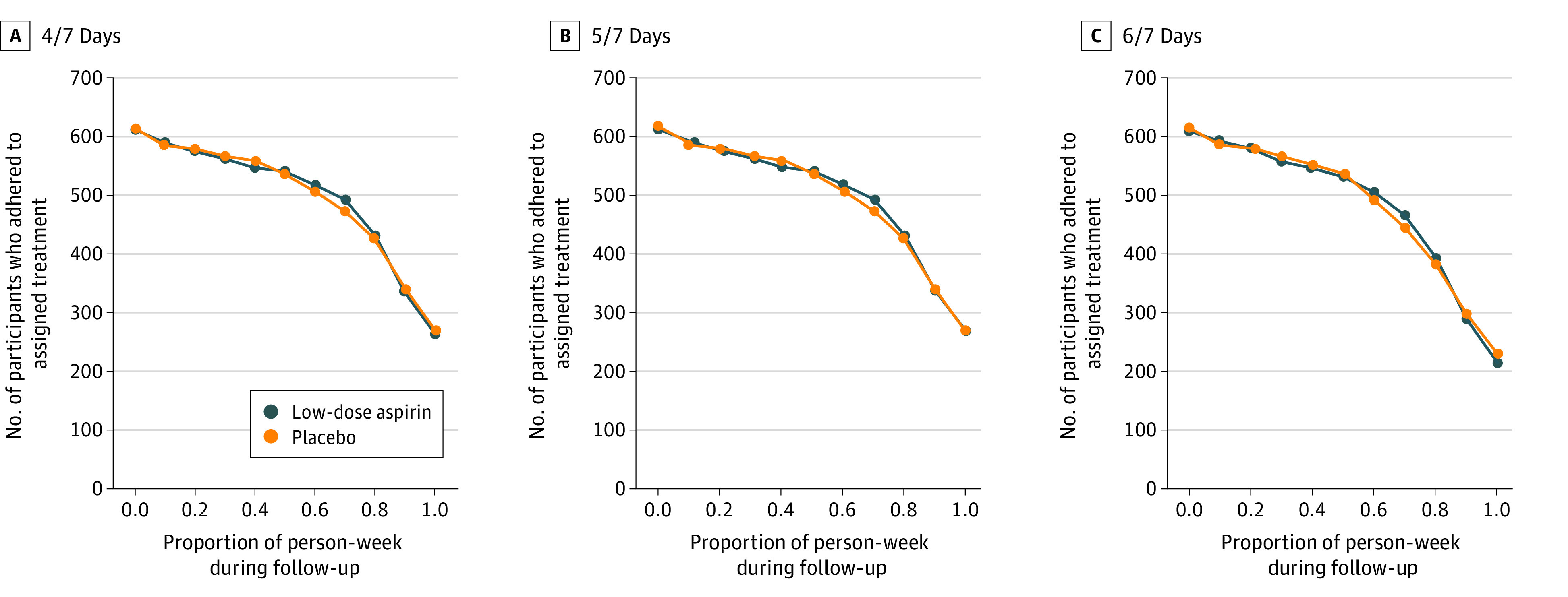
Number of Participants Who Adhered to Assigned Treatment by Different Follow-up Thresholds

A total of 1227 women were included in the analysis (mean [SD] age, 28.74 [4.80] years]). In the EAGeR trial, most of the participants were non-Hispanic White (1161 [94.6%]), had at least a high-school education (1057 [86.1%]), and were married (1123 [91.5%]) and employed (919 [74.9%]). Table 1 shows the randomized treatment assignment, outcome, baseline characteristics, and postrandomization confounders by adherence status. The CONSORT flow diagram for the EAGeR trial is presented in Figure 1. Taking at least 5 of 7 pills in a given week during at least 80% of person-weeks of follow-up was associated with the hCG-detected pregnancy outcome (χ^2^_1_ = 278.6; *P* < .001) as well as non-Hispanic White race and ethnicity (χ^2^_1_ = 17.5; *P* < .001), high school education (χ^2^_1_ = 8.2; *P* = .004), marital status (χ^2^_1_ = 33.1; *P* < .001), annual income (χ^2^_1_ = 20.1; *P* < .001), and history of smoking in the past year (χ^2^_1_ = 22.8; *P* < .001) but not with the randomized treatment assignment. Figure 2 presents the number of participants who adhered to the protocol, which decreased as the adherence threshold increased. Overall, 858 (69.9%) of the 1227 trial participants adhered to their assigned study medication protocol, and 784 (63.9%) became pregnant.

**Table 1.** Treatment Assignment, Outcome, Baseline Covariates, and Postrandomization Confounders

Variable	Adherence (n = 1227)^a^		Statistical analysis	*P* value
	No (n = 369 [30.1%])	Yes (n = 858 [69.9%])		
Treatment: daily low-dose aspirin	190 (51.5)	425 (49.5)	χ^2^_1_ = 0.4	.53
Outcome: hCG-detected pregnancy	107 (29.0)	677 (78.9)	χ^2^_1_ = 278.6	<.001
Baseline covariates				
Non-Hispanic White race	334 (90.5)	827 (96.4)	χ^2^_1_ = 17.5	<.001
High school educational level	302 (81.8)	755 (88.0)	χ^2^_1_ = 8.2	.004
Married	312 (84.6)	811 (94.5)	χ^2^_1_ = 33.1	<.001
Employed	283 (76.7)	636 (74.1)	χ^2^_1_ = 0.9	.34
Annual income ≥$40 000	213 (57.7)	608 (70.9)	χ^2^_1_ = 20.1	<.001
Exercise per week				
Low	106 (28.7)	216 (25.2)	χ^2^_2_ = 2.3	.32
Moderate	140 (37.9)	360 (42.0)		
High	123 (33.3)	282 (32.9)		
No. of previous pregnancy losses				
1	125 (33.9)	278 (32.4)	χ^2^_1_ = 0.3	.61
2	244 (66.1)	580 (67.6)		
No. of previous live births				
0	173 (46.9)	352 (41.0)	χ^2^_3_ = 6.0	.11
1	125 (33.9)	308 (35.9)		
2	68 (18.4)	179 (20.9)		
3	3 (0.8)	19 (2.2)		
Alcohol ever consumed in past year	137 (37.1)	271 (31.6)	χ^2^_1_ = 3.6	.06
Tobacco ever smoked in past year	71 (19.2)	81 (9.4)	χ^2^_1_ = 22.8	<.001
Age, median (IQR), y	28.0 (24.6-31.8)	28.4 (25.5-31.8)	NA	.12
Time attempting pregnancy before randomization, median (IQR), mo	3.0 (1.0-7.0)	3.0 (1.0-6.8)	NA	.09
BMI, median (IQR)	25.2 (21.8-30.3)	24.4 (21.3-29.3)	NA	.05
hsCRP level, median (IQR), mg/dL	0.13 (0.06-0.36)	0.11 (0.05-0.31)	NA	.22
Postrandomization confounders				
Unusual bleeding^b^	67 (18.2)	166 (19.3)	0.2 (1)	.63
Nausea and/or vomiting^c^	69 (18.7)	138 (16.1)	1.3 (1)	.26

Abbreviations: BMI, body mass index (calculated as weight in kilograms divided by height in meters squared); hCG, human chorionic gonadotropin; hsCRP, high-sensitivity C-reactive protein; NA, not applicable.

SI conversion factor: To convert hsCRP to mg/L, multiply by 10.

^a^ Adherence was measured as taking 5 of 7 pills (70%) per week for 80% of person-weeks of follow-up. Unless otherwise indicated, data are expressed as number (%) of patients.

^b^ Indicates bleeding ≥1 of 7 days (20%) per week for ≥50% of person-weeks of follow-up.

^c^ Indicates nausea and/or vomiting ≥1 of 7 days (20%) per week for ≥20% of person-weeks of follow-up.

The estimated per-protocol effect of low-dose aspirin on hCG-detected pregnancy is shown in Table 2. Relative to participants adhering to placebo, those participants who adhered to the low-dose aspirin treatment protocol experienced 8.0 (95% CI, 2.5-13.6) more hCG-detected pregnancies per 100 women in the sample, which was approximately double the ITT estimate of 4.3 (95% CI, −1.1 to 9.6) more hCG-detected pregnancies per 100 women in the sample. Risk ratios for the estimated per-protocol effects are also presented in Table 2.

**Table 2.** Effects of Low-Dose Aspirin on hCG-Detected Pregnancy Among Women With Adherence to the Assigned Treatment^a^

Method	Machine learning	Risk difference estimate (SE) [95% CI]	Risk ratio estimate (SE) [95% CI]
Intention-to-treat analysis	No	0.04 (0.03) [−0.01 to 0.10]	1.07 (0.04) [0.98 to 1.16]
Per-protocol analysis adjusted for baseline covariates			
AIPW	Yes	0.08 (0.03) [0.03 to 0.14]	1.11 (0.04) [1.03 to 1.19]
TMLE	Yes	0.08 (0.03) [0.03 to 0.13]	1.10 (0.03) [1.04 to 1.17]
g-Computation	No	0.07 (0.03) [0.02 to 0.13]	1.10 (0.03) [1.02 to 1.17]
IPW	No	0.07 (0.03) [0.02 to 0.13]	1.10 (0.04) [1.02 to 1.18]
Unadjusted per-protocol analysis	No	0.08 (0.03) [0.03 to 0.14]	1.11 (0.04) [1.04 to 1.20]

Abbreviations: AIPW, augmented inverse probability weighting; hCG, human chorionic gonadotropin; IPW, inverse probability weighting; TMLE, targeted maximum likelihood estimation.

^a^ Assigned treatment was taking 5 of 7 pills (70%) per week during at least 80% of person-weeks of follow-up.

Using other estimation methods, the per-protocol estimates remained similar, including the unadjusted estimates (Table 2 and eTable and eFigure in Supplement 2). Similar per-protocol effect estimates were also observed when adjusting for unusual bleeding and nausea and/or vomiting (risk difference per 100 woment, 8.4 [95% CI, 2.8-14.0]) (eTable in Supplement 2). Using other adherence thresholds, our sensitivity analyses with AIPW and machine learning show per-protocol effect estimates increase. These per-protocol effect estimates ranged from 5.6 per 100 women (95% CI, 0.0-11.2) to 9.0 per 100 women (95% CI, 3.4-14.5) when adherence thresholds ranged from 4 of 7 days for at least 60% of person-weeks of follow-up to 6 of 7 days for at least 80% of person-weeks of follow-up (eFigure in Supplement 2).

## Discussion

We demonstrate the use of stacked machine learning with AIPW in estimating the per-protocol effects in the EAGeR trial. Our time-fixed, per-protocol analysis results were consistent with previous findings of the per-protocol effect estimate of aspirin that accounted for the time-varying nature of adherence and select time-varying confounders.^4^ However, unlike previous research, we used nonparametric machine learning methods to estimate these effects. Our analyses demonstrate a novel approach for per-protocol effect estimation using advanced statistical methods. In addition, our results suggest that a preconception low-dose aspirin regimen increases hCG-detected pregnancies for women with 1 or 2 prior pregnancy losses who adhered to at least 5 of 7 days of low-dose aspirin therapy for at least 80% of the follow-up.

Supervised machine learning algorithms have been widely adopted to predict various health outcomes.^33,34,35^ Although they can also be used for effect estimation, additional steps are needed.^13,14^ Importantly, these steps nclude the need to adjust for relevant confounders and to use doubly robust methods such as AIPW.

The benefits of using machine learning with doubly robust methods lie primarily in the ability to avoid strong parametric modeling assumptions. Machine learning models can be more flexible and data adaptive than traditional regression models.^15,16^ For example, the inclusion of an interaction term in a regression model is determined by the investigators’ domain-specific knowledge, whereas tree-based models (eg, random forests) adopt a more data-adaptive approach to interaction inclusion.^36,37^ Failure to include an interaction term may result in model misspecification and lead to biased effect estimation. However, as a result of this increased data adaptiveness and extra modeling flexibility, tree-based models—and flexible machine learning in general—are more likely to overfit the data and have larger mean squared error.^36^ To mitigate these issues, combining tree-based methods (eg, random forests) and regression-based methods (eg, generalized linear models and multivariate adaptive regression splines) is advisable.^13,16^ In our study, we stacked 5 different machine learning models to create added flexibility and used cross-validation to mitigate overfitting.

The purpose of this study was to illustrate the use of machine learning as an alternative approach for per-protocol effect estimation rather than convincing the readers to use machine learning only. However, it should be recognized that if the model is correctly specified (even this is a rare scenario), parametric regression is more statistically efficient than flexible machine learning methods.^13^

We used supervised machine learning methods with doubly robust estimators to quantify the per-protocol effect of aspirin on hCG-detected pregnancy. In an RCT where all participants are fully adherent with the treatment protocol, per-protocol effect will be identical to ITT effects.^38^ However, in the EAGeR trial, the ITT effects of low-dose aspirin on hCG-detected pregnancy differed substantially from the estimated per-protocol effect owing to nonadherence with the specified protocol during follow-up. In many settings captured by clinical trials with repeated opportunities to take the assigned treatment (eg, every day during weeks of follow-up), perfect adherence is unlikely, and a practical adherence level has to be chosen based on either clinical knowledge or the data at hand. In the EAGeR trial, the adherence rate declined over time and dropped quickly after the start of pregnancy.^4^ We defined adherence based on a protocol of taking 5 of 7 pills in a given week for at least 80% of person-weeks because some literature suggests that a biological effect of low-dose aspirin could be achieved at this adherence level^4^ and because of the relatively short half-life of aspirin.^39^

We found that our unadjusted estimates were similar to estimates we obtained by improperly adjusting for postrandomization confounders (eg, unusual bleeding and nausea and/or vomiting) but properly adjusting for baseline confounders (eg, age, marital status, or annual income). In addition, these results aligned closely with those from a prior study^4^ that properly adjusted for postrandomization confounding, albeit with methods that were much less flexible (ie, parametric g-computation). This finding lends additional empirical support to the use of daily low-dose aspirin in increasing hCG-detected pregnancies.

Our analytic approach using time-fixed adherence can be more broadly applied in other analysis principles of RCTs as well as in observational studies, particularly those with only 1 time point. For example, our approach can be directly applied to the as-treated analysis in RCTs, such as a trial for evaluating the efficacy of emergency contraception. Modified ITT (despite not being consistently defined)^40,41^ can be incorporated with our approach as well because the modification of ITT may not be free of confounding (eg, only including participants with initiation of drug therapy for a nonblinded study). Further, adjusting for covariates with machine learning in (modified) ITT analysis can improve statistical efficiency for higher precision of treatment effect estimates.^42^

### Limitations

This study has some limitations. In a well-conducted trial, an ITT approach provides unbiased estimates of the assignment effect. The ITT estimates capture the impact of the treatment assignment strategy and generally can be interpreted as the effectiveness of recommending or prescribing one treatment compared with another.^1^ In contrast, an appropriately adjusted per-protocol analysis can be used to estimate the effect of taking the active treatment according to the specifications of the protocol, allowing estimation of the treatment efficacy. Similar to most per-protocol analyses, our study relied on time-fixed adherence status, which is an important limitation. Although our effect estimates of low-dose aspirin on hCG-detected pregnancy are similar to those of the prior study that accounted for time-varying adherence,^4^ limitations should be considered when conducting a time-fixed, per-protocol analysis. First, in conducting a time-fixed analysis, we had to collapse time-varying adherence status into a single time point, losing detailed information of how adherence changed during follow-up. Second, time-fixed analyses are generally unable to appropriately adjust for time-varying confounders, such as unusual bleeding and nausea. For example, at a given time point, adherence to treatment is associated with an increased likelihood of adverse effects (eg, unusual bleeding), which is further associated with a decreased likelihood of adherence at the next time point. Therefore, postrandomization confounders (eg, unusual bleeding and nausea) could simultaneously mediate and confound the effect of adherence status, requiring an analytic approach that we did not use.^28^ In addition, other common limitations of observational studies should also be considered in the per-protocol analysis, such as unmeasured confounders. Last, we had limited information on important variables such as race and ethnicity, which limits the generalizability of our findings.

## Conclusions

This secondary analysis of a randomized clinical trial suggests that machine learning methods with doubly robust estimators, such as AIPW, can be used to estimate per-protocol treatment effects. Furthermore, our empirical findings align with prior results supporting the prophylactic use of daily low-dose aspirin to improve the chances of hCG-detected pregnancy in women at high risk of pregnancy loss.

## Notes

Supplement 1.
Trial Protocol

Supplement 2. **eTable.** Sensitivity Analyses of the Effects of Low-Dose Aspirin on hCG-Detected Pregnancy Among Women Adhering to the Assigned Treatment: 5 of 7 Pills per Week Over at Least 80% of Person-Weeks of Follow-up Using Different Estimation Methods **eFigure.** Sensitivity Analyses of the Effects of Low-Dose Aspirin on hCG Conception Using Different Adherence Levels and Estimation Methods **eMethods.** R Code for Per-Protocol Effect Estimation

Supplement 3.
Data Sharing Statement
