# Errors in Figure 1 and Supplement and Clarification of Methods

**DOI:** 10.1001/jamanetworkopen.2022.9172

**Published:** 2022-04-14

The Original Investigation titled “Use of Machine Learning to Estimate the Per-Protocol Effect of Low-Dose Aspirin on Pregnancy Outcomes: A Secondary Analysis of a Randomized Clinical Trial,”^1^ published March 9, 2022, included an incorrect version of Figure 1 and a Supplement that contained formatting errors. In Figure 1, the CONSORT diagram omitted the numbers of patients included in the intention-to-treat analysis and listed incorrect numbers of patients included in the per-protocol analysis. In the Supplement, the eTable had misaligned column headings and the eFigure had overlapping characters in the caption. In addition, in the second to last paragraph of the Methods section, edits were made to clarify the process by which sensitivity analyses were performed. The article and Supplement have both been corrected.^1^
