# Supplementary material for: Use of Machine Learning to Estimate the Per-Protocol Effect of Low-Dose Aspirin on Pregnancy Outcomes: A Secondary Analysis of a Randomized Clinical Trial
Source: JAMA Netw Open. 2022 Mar 9;5(3):e2143414. doi: 10.1001/jamanetworkopen.2021.43414 (PMC8908068; doi:10.1001/jamanetworkopen.2021.43414)
Supplement: Supplement 2. — eTable. Sensitivity Analyses of the Effects of Low-Dose Aspirin on hCG-Detected Pregnancy Among Women Adhering to the Assigned Treatment: 5 of 7 Pills per Week Over at Least 80% of Person-Weeks of Follow-up Using Different Estimation Methods eFigure. Sensitivity Analyses of the Effects of Low-Dose Aspirin on hCG Conception Using Different Adherence Levels and Estimation Methods eMethods. R Code for Per-Protocol Effect Estimation [file jamanetwopen-e2143414-s002.pdf]

Supplementary Online Content

Zhong Y, Brooks MM, Kennedy EH, Bodnar LM, Naimi AI. Use of machine learning to estimate the per-protocol effect of low-dose aspirin on pregnancy outcomes: a secondary analysis of a randomized clinical trial. *JAMA Netw Open*. 2022;5(3):e2143414. doi:10.1001/jamanetworkopen.2021.43414

eTable. Sensitivity Analyses of the Effects of Low-Dose Aspirin on hCG-Detected Pregnancy Among Women Adhering to the Assigned Treatment: 5 of 7 Pills per Week Over at Least 80% of Person-Weeks of Follow-up Using Different Estimation Methods

eFigure. Sensitivity Analyses of the Effects of Low-Dose Aspirin on hCG Conception Using Different Adherence Levels and Estimation Methods

eMethods. R Code for Per-Protocol Effect Estimation

This supplementary material has been provided by the authors to give readers additional information about their work.

© 2022 Zhong Y et al. *JAMA Network Open*.

eTable. Sensitivity Analyses of the Effects of Low-Dose Aspirin on hCG-Detected Pregnancy Among Women Adhering to the Assigned Treatment: 5 of 7 Pills per Week Over at Least 80% of Person-Weeks of Follow-up Using Different Estimation Methods

		RD				RR			
Method	Machine Learning	Est.	SE	LCL	UCL	Est.	SE	LCL	UCL
Intention-to-treat	No	0.043	0.027	-0.011	0.096	1.069	0.043	0.982	1.163
**Per-protocol analysis adjusted for baseline covariates**									
AIPW	Yes	0.080	0.028	0.025	0.136	1.107	0.036	1.032	1.188
TMLE	Yes	0.078	0.025	0.030	0.126	1.104	0.031	1.038	1.174
G-computation	No	0.073	0.027	0.019	0.125	1.097	0.035	1.024	1.173
IPW	No	0.075	0.028	0.019	0.130	1.099	0.036	1.024	1.180
**Per-protocol analysis adjusted for baseline covariates and post-randomization confounders***									
AIPW	Yes	0.084	0.029	0.028	0.140	1.113	0.037	1.036	1.196
TMLE	Yes	0.078	0.025	0.030	0.126	1.104	0.031	1.038	1.174
G-computation	No	0.073	0.027	0.020	0.125	1.097	0.034	1.024	1.172
IPW	No	0.073	0.028	0.018	0.128	1.097	0.036	1.022	1.177
**Unadjusted per-protocol analysis**									
Unadjusted	No	0.078	0.028	0.023	0.132	1.103	0.035	1.029	1.183

AIPW: Augmented Inverse Probability WeightingTMLE: Targeted Maximum Likelihood EstimationIPW: Inverse Probability Weighting

* Adjusted for unusual bleeding [$\geq 1/7 days (20\%)$ per week over ≥ 50% person-week] and nausea and/or vomiting [$\geq 1/7 days (20\%)$ per week over ≥ 20% person-week]. **CAUTION: adjusting for post-randomization confounders in a time-fixed setting (i.e., collapsing time-varying information into a dichotomous variable) is inappropriate.**

© 2022 Zhong Y et al. *JAMA Network Open*.

eFigure. Sensitivity Analyses of the Effects of Low-Dose Aspirin on hCG Conception Using Different Adherence Levels and Estimation Methods

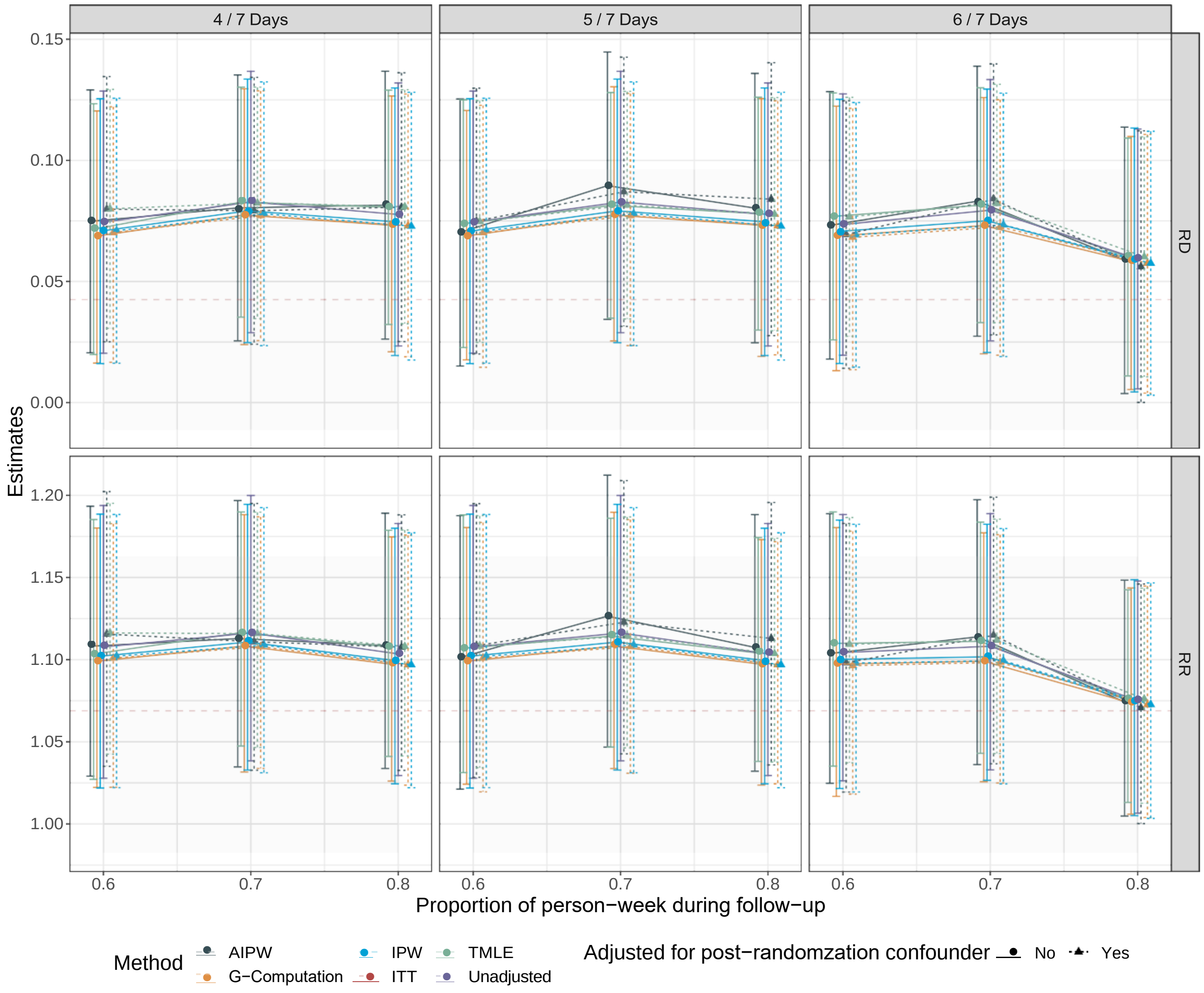

AIPW: Augmented Inverse Probability Weighting
TMLE: Targeted Maximum Likelihood Estimation
IPW: Inverse Probability Weighting
Post-randomization confounder: unusual bleeding [$\geq 1/7 days$(20%) per week over ≥50% person-week] and nausea and/or vomiting[1≥7*days*(20%) per week over 20%≥person-week]. **CAUTION: adjusting for post-randomization confounders in a time-fixed setting (i.e., collapsing time-varying information into a dichotomous variable) is inappropriate.**

© 2022 Zhong Y et al. *JAMA Network Open*.

eMethods. R code for Per-Protocol Effect Estimation

Setup

Load necessary packages and dataset

```
packages <- c("tidyverse", "AIPW", "SuperLearner",
              "earth","ranger","xgboost")

for (package in packages) {
  if (!require(package, character.only=T, quietly=T)) {
    install.packages(package,repos='http://lib.stat.cmu.edu/R/CRAN')
  }
}

#read dataset
eager_analysis <- read_csv("eager_pp_df_20200615.csv")
```

Intention-to-treat

```
# unadjusted estimates
get_all_est <- function(x){
  mat <- as.matrix(x)
  p1 <- mat[2,2]/sum(mat[2,])
  p0 <- mat[1,2]/sum(mat[1,])
  res <- data.frame(
    #Est./ SE
    RD = c(p1-p0,
           sqrt(p1*(1-p1)/sum(mat[2,])+p0*(1-p0)/sum(mat[1,]))),
    logRR = c( log(p1/p0),
               sqrt((1-p1)/mat[2,2]+(1-p0)/mat[1,2])),
    logOR = c( log((p1/(1-p1))/(p0/(1-p0))),
               sqrt(1/mat[2,2]+1/mat[1,2]+1/mat[1,1]+1/mat[2,1]))
  ) %>%
    rbind(., .[1,] - 1.96 * .[2,], .[1,] + 1.96 * .[2,]) %>%
    bind_cols(N = sum(mat),
              Param = c("Est.","SE","LCL","UCL"),
              Method = "Unadj")
  return(res)
}
#ITT estimates
itt <- get_all_est(table(eager_analysis$treatment,eager_analysis$conception))
itt
```

Per-protocol

Machine Learning + AIPW Adjusting for Baseline Covariates

```
# Set seeds
set.seed(123)
# Define learners for stacking machine learning via SuperLearner
earth_learner   <- create.Learner("SL.earth",tune=list(degree=c(2,3)))
ranger_learner  <- create.Learner("SL.ranger", tune=list(min.node.size = c(30),
                                                          num.trees=c(500),
                                                          max.depth=c(2,3)))
xgboost_learner <- create.Learner("SL.xgboost", tune=list(minobspernode = c(30),
```

© 2022 Zhong Y et al. *JAMA Network Open*.

```
                                                              ntrees=c(500),
                                                              max_depth=c(2,3),
                                                              subsample=c(1)))

sl.lib <- c("SL.glm", "SL.glm.interaction",
            earth_learner$names,ranger_learner$names, xgboost_learner$names)

# Subset dataset to those adhered to the protocol
df <- eager_analysis %>% filter(weeks_0.7_pt_0.8==1)

# select baseline covariates
bl_cov <- df %>%
  select(income_1:hsCRP) %>%
  as.data.frame()

# AIPW estimation via AIPW package
aipw_fit1 <- AIPW$new(Y=df$conception,
                      A=df$treatment,
                      W=bl_cov,
                      g.SL.library = sl.lib,
                      Q.SL.library = sl.lib,
                      k_split = 10,
                      verbose = TRUE)$
  fit()$
  summary(g.bound=0.025)$
  # check positivity
  plot.p_score()
```

Machine Learning + AIPW Adjusting for Baseline Covariates + Post-randomization Confounders

```
# select post-randomization confounders
postRand_confounder <- df %>%
  select(bleed_0.2_pt_0.5,nausea_0.2_pt_0.2) %>%
  as.data.frame()

# AIPW estimator via AIPW package
aipw_fit2 <- AIPW$new(Y=df$conception,
                      A=df$treatment,
                      W=cbind(bl_cov,postRand_confounder),
                      g.SL.library = sl.lib,
                      Q.SL.library = sl.lib,
                      k_split = 10,
                      verbose = TRUE)$
  fit()$
  summary(g.bound=0.025)$
  # check positivity
  plot.p_score()
```

© 2022 Zhong Y et al. *JAMA Network Open*.
