# Supplementary material for: Use of Machine Learning to Estimate the Per-Protocol Effect of Low-Dose Aspirin on Pregnancy Outcomes: A Secondary Analysis of a Randomized Clinical Trial
Source: JAMA Netw Open. 2022 Mar 9;5(3):e2143414. doi: 10.1001/jamanetworkopen.2021.43414 (PMC8908068; doi:10.1001/jamanetworkopen.2021.43414)
Supplement: Supplement 3. — Data Sharing Statement [file jamanetwopen-e2143414-s003.pdf]

# Data Sharing Statement

Zhong. Use of Machine Learning to Estimate the Per-Protocol Effect of Low-Dose Aspirin on Pregnancy Outcomes. *JAMA Netw Open*. Published March 09, 2022.
doi:10.1001/jamanetworkopen.2021.43414

**Data**
**Data available:** No

**Additional Information**
**Explanation for why data not available:** Trial data will be made accessible in an electronic repository after completion of the study's analytical phases. The data, along with a set of guidelines for researchers applying for use of the data, will be posted to a data sharing site: Eunice Kennedy Shriver National Institute of Child Health and Human Development Data and Specimen Hub (https://dash.nichd.nih.gov).
